# New Antibacterial Secondary Metabolites from a Marine-Derived *Talaromyces* sp. Strain BTBU20213036

**DOI:** 10.3390/antibiotics11020222

**Published:** 2022-02-10

**Authors:** Fuhang Song, Yifei Dong, Shangzhu Wei, Xinwan Zhang, Kai Zhang, Xiuli Xu

**Affiliations:** 1 School of Light Industry, Beijing Technology and Business University, Beijing 100048, China; songfuhang@btbu.edu.cn (F.S.); dongyifei1930201016@st.btbu.edu.cn (Y.D.); zhangkai2030302071@st.btbu.edu.cn (K.Z.); 2 School of Ocean Sciences, China University of Geosciences, Beijing 100083, China; 2111180016@cugb.edu.cn (S.W.); zhangxinwan@cugb.edu.cn (X.Z.)

**Keywords:** marine-derived fungus, *Talaromyces* sp., antibacterial, *Staphylococcus aureus*, polyketide

## Abstract

New polyketide-derived oligophenalenone dimers, bacillisporins K and L (**1** and **2**) and xanthoradone dimer rugulosin D (**3**), together with four known compounds, bacillisporin B (**4**), macrosporusone D (**5**), rugulosin A and penicillide (**6** and **7**), were isolated from the marine-derived fungus *Talaromyces* sp. BTBU20213036. Their structures were determined by detailed analysis of HRESIMS, 1D and 2D NMR data, and the absolute configurations were determined on the basis of calculated and experimental electronic circular dichroism (ECD). The antibacterial and antifungal activities of these compounds were tested against Gram-positive—*Staphylococcus aureus*, Gram-negative—*Escherichia coli*, and fungal strain—*Candida albicans*. These compounds showed potential inhibitory effects against *S. aureus* with minimum inhibitory concentrations ranging from 0.195 to 100 µg/mL.

## 1. Introduction

The discovery and introduction into clinics of antibiotics have made great contributions to human health. The most widely applied antibiotics in clinics were discovered in the period between the 1950s and 1960s, while the overuse of antibiotics lead to the emergence of drug-resistant bacteria, which is recognized globally by scientists nowadays [1,2]. The spread of multi-drug resistant microorganisms is getting more and more serious to humans [3,4,5]. It is estimated that 700,000 people died as a result of infections caused by antimicrobial resistant bacteria annually [6]. There is an urgent demand to screen new antibiotics in order to combat the infective diseases caused by drug-resistant bacteria.

**Figure 1:**
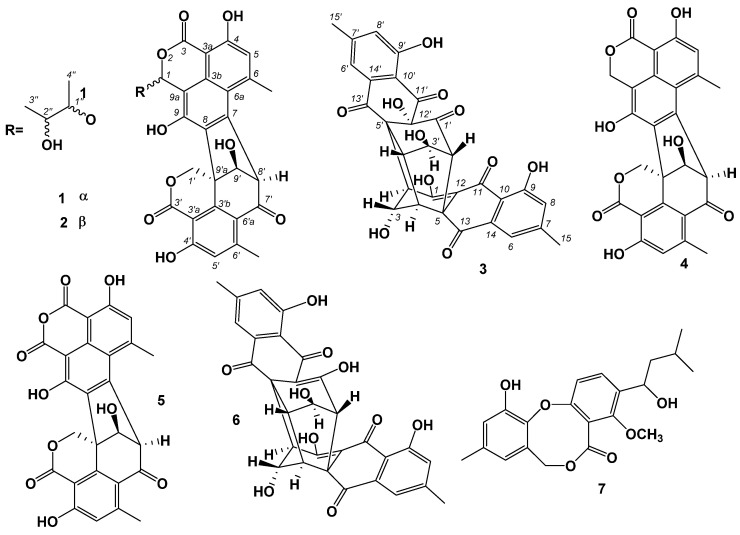
Chemical structures of **1**–**7**.

Fungi from marine environments have proven to be important pools for structurally unique and biologically diverse natural products [7]. *Talaromyces* fungi belong to ascomycetous. A number of *Talaromyces* species have been isolated from marine environments, such as, *Talaromyces albobiverticillius*, *Talaromyces assiutensis*, *Talaromyces purpureogenus* [8,9,10,11,12]. Fungi of *Talaromyces* genus isolated from marine environments produced a series of bioactive natural products, such as oligophenalenones [11,12,13,14,15], terpenoids [16,17], naphthoquinones [18], spolyene and isocoumarin [19], diphenyl ether derivatives, sesquiterpene-conjugated amino acids [20,21], lactones [22], and ergosterol analog and bisanthraquinone [23]. In the course of our continuing investigation of bioactive natural products from marine-derived fungi [24,25,26], the fungal strain *Talaromyces* sp. BTBU20213036, which was obtained from a mud sample collected from the coastal region of Qingdao, Shandong Province, exhibited antimicrobial activity against *Staphylococcus aureus*. Fermentation scale-up of this strain was conducted in rice solid media. The study of the chemical constituents of the fermentation materials resulted in the isolation and characterization of three new secondary metabolites, including bacillisporins K and L (**1** and **2**) and rugulosin D (**3**), together with four known compounds, bacillisporin B [15], macrosporusone D [27], rugulosin A [28] and penicillide [29] (Figure 1). **1**, **2** and **4**−**6** showed potential antibacterial activities against *S. aureus*. Herein we report the details of isolation, structure elucidation, and antimicrobial activities evaluation of these compounds.

## 2. Results

**Figure 2:**
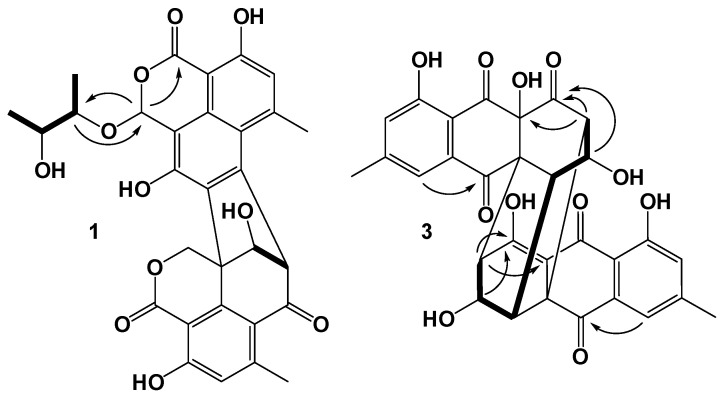
Key ^1^H-^1^H COSY and HMBC correlations for **1** and **3**.

Compound **1** was isolated as a light yellow amorphous powder. The molecular formula of **1** was determined to be C_30_H_26_O_11_ based on high resolution electrospray ionization mass spectrum (HRESIMS) (*m/z* [M + H]^+^ 585.1374, calcd for C_30_H_27_O_11_, 585.1367), accounting for eighteen degrees of unsaturation (Figure S1). Figure S2 showed the High Performance Liquid Chromatography (HPLC) profile and ultraviolet (UV) spectrum of **1.** The ^1^H, ^13^C and Heteronuclear Single Quantum Correlation (HSQC) spectra of **1** (Figures S3–S5, Table 1) showed the presence of four methyl groups [*δ*_H_ 2.98/*δ*_C_ 24.4 (6-Me), *δ*_H_ 2.48/*δ*_C_ 23.2 (6′-Me), *δ*_H_ 0.75/*δ*_C_ 15.6 (C-3″), *δ*_H_ 0.99/*δ*_C_ 17.2 (C-4″)], one oxygenated methylene group [*δ*_H_ 5.12 and 4.95/*δ*_C_ 69.9 (C-1′)], five sp^3^ methine groups [*δ*_H_ 6.86/*δ*_C_ 98.8 (C-1), *δ*_H_ 4.83/*δ*_C_ 64.9 (C-8′), *δ*_H_ 4.77/*δ*_C_ 85.1 (C-9′), *δ*_H_ 4.12/*δ*_C_ 78.6 (C-1″), *δ*_H_ 3.72/*δ*_C_ 68.9 (C-2″)], two aromatic methines [*δ*_H_ 6.96/*δ*_C_ 118.4 (C-5), *δ*_H_ 6.83/*δ*_C_ 119.6 (C-5′)], one sp^3^ quaternary carbon [*δ*_C_ 49.5 (C-9′a)], as well as seventeen sp^2^ quaternary carbons including one ketone carbonyl [*δ*_C_ 192.5 (C-7′)] and two lactone carboxyls [*δ*_C_ 168.3 (C-3), 167.8 (C-3′)]. ^1^H-^1^H Correlation Spectroscopy (COSY) spectrum (Figure 2 and Figures S6) indicated the side chain of C-3″/C-2″/C-1″/C-4″. By comparing the NMR data with those of **5**, one of the lactones was replaced by the acetal methine [*δ*_H_ 6.86 (s)/*δ*_C_ 98.8 (C-1)] and the linkage between C-1 and C-1″ through the oxygen atom was confirmed by Heteronuclear Multiple Bond Correlation (HMBC) correlations (Figure 2 and Figure S7, Table S1) from H-1 to C-3 and C-1″ and from H-1″ to C-1. The structure of **1** was further established by ^1^H-^1^H COSY (Figure 2 and Figure S6) and HMBC experiments. The relative configurations were deduced by the singlet peaks for H-8′ [*δ*_H_ 4.83 (s)] and H-9′ [*δ*_H_ 4.77 (brs)] and Rotating Frame Overhauser Spectroscopy (ROESY) correlation between H-8′ and H-9′ (Figure S8).

Compound **2** was isolated as a light yellow amorphous powder. The molecular formula of **2** was determined to be C_30_H_26_O_11_ based on the HRESIMS spectrum (*m/z* [M + H]^+^ 585.1377, calcd for C_30_H_27_O_11_, 585.1367), accounting for eighteen degrees of unsaturation (Figure S9). Figure S10 showed the HPLC profile and UV spectrum of **2**. The ^1^H, ^13^C and HSQC spectra of **1** (Figures S11–S13, Table 1) showed similar data to those of **2**. The differences are signals for acetal methine [*δ*_H_ 6.81/*δ*_C_ 99.6 (C-1)] and the substructure attached to C-1 [*δ*_H_ 3.90/*δ*_C_ 80.2 (C-1″), 3.61/*δ*_C_ 71.3 (C-2″), 1.14/*δ*_C_ 18.9 (C-3″), 1.11/*δ*_C_ 17.9 (C-4″)]. These data revealed the configurations of C-1, C-1″, C-2″ were different from those of **2**, which resulted in the different deshielding effects from the aromatic moiety. Furthermore, the structure was characterized by detailed analysis of 2D NMR spectra (Figure 2 and Figures S13–S15). In the REOSY spectrum (Figure S16), the crossing peaks between H-8′ and H-9′-OH, and between H-9′ and H-1′a revealed the relative configurations of C-8′, 9′ and C-9a′ (Table S2).

**Figure 3:**
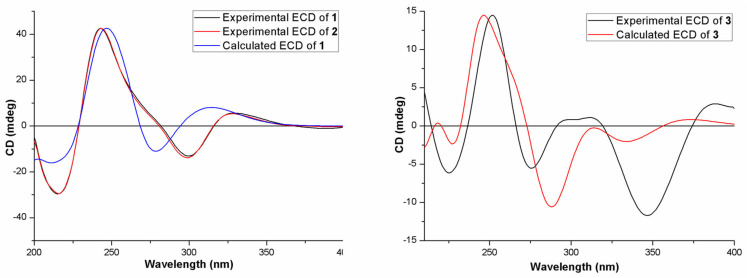
Experimental and calculated ECD spectra of **1**, **2** and **3**.

Compounds **1** and **2** showed almost the same experimental ECD spectra, which were consistent with the reported bacillisporin I [15] and calculated data (Figure 3). Thus, the configurations of **1** and **2** were determined as 8′*R*, 9′*S*, 9′a*S***,** while the configurations of C-1, C-1″ and C-2″ were not determined. Compounds **1** and **2** were named bacillisporins K and L, respectively.

**Figure 4:**
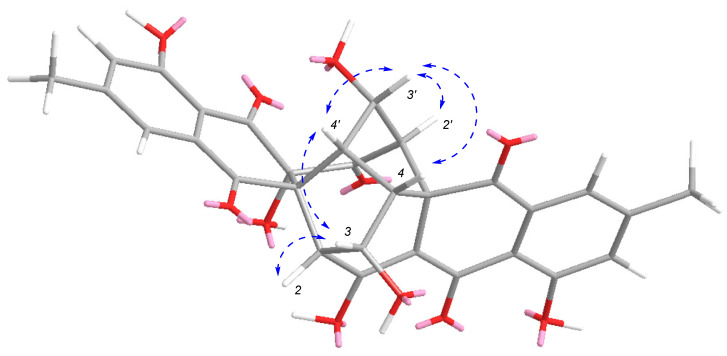
Key ROESY correlations of **3**.

Compound **3** was isolated as a brown amorphous powder. The molecular formula of **3** was determined to be C_30_H_22_O_11_ based on the HRESIMS spectrum (*m/z* [M + H]^+^ 559.1234, calcd for C_30_H_23_O_11_, 559.1235), accounting for twenty degrees of unsaturation (Figure S17). Figure S18 showed the HPLC profile and UV spectrum of **3**. The ^1^H, ^13^C, HSQC and ^1^H- 1H COSY spectra of **2** (Figures S19–S22, Table 2) showed signals for two singlet methyl groups [*δ*_H_ 2.44/*δ*_C_ 21.6 (C-15), *δ*_H_ 2.43/*δ*_C_ 21.5 (C-15′)], six sp^3^ methine groups [*δ*_H_ 2.73/*δ*_C_ 55.7 (C-2), *δ*_H_ 2.90/*δ*_C_ 63.4 (C-2′), *δ*_H_ 4.27/*δ*_C_ 70.2 (C-3), *δ*_H_ 4.56/*δ*_C_ 69.0 (C-3′), *δ*_H_ 3.46/*δ*_C_ 48.1 (C-4), *δ*_H_ 3.73/*δ*_C_ 44.0 (C-4′)], four aromatic methines [*δ*_H_ 7.46/*δ*_C_ 120.6 (C-6), *δ*_H_ 7.41/*δ*_C_ 120.1 (C-6′), 7.24/*δ*_C_ 124.0 (C-8), *δ*_H_ 7.21/*δ*_C_ 123.8 (C-8′)], three sp^3^ quaternary carbons including one oxygenated carbons [*δ*_C_ 74.6 (C-12′)], five ketone carbonyls [[*δ*_C_ 198.8 (C-1′), 184.8 (C-11), [*δ*_C_ 192.1 (C-11′), 193.0 (C-13), 192.8 (C-13′)], as well as ten sp^2^ quaternary carbons [*δ*_C_ 178.1 (C-1), 148.5 (C-7)/148.6 (C-7′), 160.9 (C-9)/161.0 (C-9′), 114.3 (C-10)/113.3 (C-10′), 106.8 (C-12), 132.3 (C-14)/133.5 (C-14′)]. By comparing the NMR data with those of rugulosin A [28], the structure was deduced as an analogue of rugulosin A (**6**). Detailed analysis of the NMR data revealed that the sp^2^ quaternary carbons of C-1′ [*δ*_C_ 186.7] and C-12′ [*δ*_C_ 106.8] in rugulosin A were replaced by one ketone carbonyl [*δ*_C_ 198.8] and one oxygenated sp^3^ quaternary carbon [*δ*_C_ 74.6]. The planar structure of **3** further confirmed by HMBC correlations (Figure 2 and Figure S23, Table S3) from H-2′ to C-1′ and C-12′. The relative configurations of **3** were deduced by comparing the literature data for ^1^H NMR between rugulosin A and **3**, the chemical shifts of H-3/H-3′ of **3** were *δ*_H_ 4.27/4.56 with a coupling constant of 5.0 and 4.5 Hz, which were almost the same as those reported for rugulosin A [28]. In the ROESY spectrum (Figure 4 and Figure S24), correlations between H-3′ and H-2′, H-4/H-4′, and between H-3 and H-2 and H-4′ were observed, which confirmed the relative stereochemistry of **3**. By comparison of experimental and calculated ECD spectra (Figure 4), the absolute configurations of **3** were determined as shown in Figure 1 and named rugulosin D.

Four known compounds, bacillisporin B [15], macrosporusone D [27], rugulosin A [28] and penicillide [29] were also isolated and characterized by comparing their molecular weight and NMR data with those reported in the literature.

These compounds were tested for antibacterial activities against a panel of pathogens of *S. aureus* ATCC 25923, *Escherichia coli* ATCC 25923 and *Candida albicans* ATCC 10231. Compounds **1**, **2** and **4**−**6** strongly inhibited the growth of *S. aureus* with MIC values of 12.5, 25, 12.5, 6.25, and 0.195 µg/mL (Table 3). None of the tested compounds showed inhibitory effects against *C. albicans* and *E. coli* at concentration of 100 µg/mL.

## 3. Materials and Methods

### 3.1. General Experimental Procedures

Optical rotations ([α]_D_) were measured by using an Anton Paar MCP 200 Modular Circular Polarimeter (Austria) in a 100 × 2 mm cell at 25 °C. CD spectra were recorded on Applied Photophysics Chirascan spectropolarimeter (Surrey, UK). NMR experiments were carried on a Bruker Avance 500 spectrometer at 25 °C with residual solvent peaks as references (DMSO-*d*_6_: *δ*_H_ 2.50, *δ*_C_ 39.52). High resolution ESIMS spectra were measured using an Accurate-Mass-Q-TOF LC/MS 6520 instrument (Santa Clara, CA, USA) in positive ion mode. HPLC was run on an Agilent 1200 Series instrument.

### 3.2. Microbial Material

Strain BTBU20213036 was isolated from a mud sample collected from the intertidal zones of the Yellow Sea in Qingdao, China, and grown on a potato dextrose agar plate at 28 °C for 10 days. Colonies were about 25 mm diam, texture floccose and funiculose, sporulation abundant, dark greyish green, mycelium yellow, no exudate and soluble pigment, colony reverse brown (Figure S25). The genomic DNA of BTBU20213036 was extracted using DNAquick Plant System (Tiangen, Beijing, China). The ITS sequence of BTBU20213036 was amplified by using a conventional primer pair of ITS4 (5′-TCCTCCGCTTATTGATATGC-3′) and ITS5 (5′-GGAAGTAAAAGTCGTAACAAGG-3′). PCR products were sequenced by Beijing Qingke Biotechnology Co., Ltd. (Beijing, China) and the sequence was deposited in GenBank (accession number, OM049426). Strain BTBU20213036 was identified as *Talaromyces* sp. based on gene sequence analysis of ITS by comparing with sequences from GenBank database using BLAST program (Figure S26). Alignments and calculations of sequence similarity were carried out using CLUSTAL W [30]. The strain was deposited in Beijing Technology and Business University, Beijing, China.

### 3.3. Fermentation, Extraction and Purification

*Talaromyces* sp. BTBU20213036 was inoculated on a potato dextrose agar plate and incubated at 28 °C for 7 days. A slice of fungal colony of 1 cm^2^ was put into twenty of 1 L conical flasks, each containing 200 g of raw rice, which was soaked in distilled water for 60 min. The inoculated flasks were incubated stationary at 28 °C for 30 days. The fermented materials of *Talaromyces* sp. BTBU20213036 were extracted three times by EtOAc:MeOH (80:20), and the organic solvent was evaporated in vacuo at 45 °C to yield brown crude extract (18.4 g). The crude extract was resuspended into 500 mL distilled water and extracted by 500 mL EtOAc (three times). Then EtOAc was evaporated *in vacuo* at 45 °C to give a dark residue (5.91 g). The EtOAc extract was separated by a reduced pressure silica gel chromatography (50 × 80 mm column, TLC H silica) with a stepwise gradient of 80–100% hexane/CH_2_Cl_2_ and then 0–90% MeOH/CH_2_Cl_2_ to afford 15 fractions. The eighth fraction was purified on a Sephadex LH-20 column using an elution of CH_2_Cl_2_:MeOH (2:1) to give four subfractions. The third subfraction was further separated by HPLC (Agilent ZORBAX SB-C18, 250 × 9.4 mm, 5 μm column, 3.0 mL/min) eluting with 40 – 50% MeCN/H_2_O in 15 min, then to 82% MeCN/H_2_O in 20 min to yield compounds **7** (10.2 mg), **5** (3.4 mg), **1** (4.9 mg), **4** (1.2 mg) and **2** (3.4 mg). The ninth fraction was subjected to a Sephadex LH-20 chromatography eluting by CH_2_Cl_2_:MeOH (2:1) to give four subfractions. The third subfraction was further purified by HPLC (Agilent ZORBAX Eclipse SB-C18, 250 × 9.4 mm, 5 μm column, 3.0 mL/min) eluting by 70% MeOH/H_2_O to give compounds **3** (11.2 mg) and **6** (8.4 mg). The procedure for extraction and compounds isolation was shown in Figure S27. 

#### 3.3.1. Bacillisporin K (**1**)

Bacillisporin K (**1**): Light yellow amorphous powder; ${\left[\alpha \right]}_{D}^{25}$ +206.0 (*c* 0.1, MeOH); ^1^H and ^13^C NMR data, Table 1; HRESIMS *m/z* 585.1374 [M + H]^+^ (calcd for C_30_H_27_O_11_, 585.1367).

#### 3.3.2. Bacillisporin M (**2**)

Bacillisporin L (**2**): Light yellow amorphous powder; +231.5 (*c* 0.2, MeOH); ^1^H and ^13^C NMR data, Table 1; HRESIMS *m/z* 585.1377 [M + H]^+^ (calcd for C_30_H_27_O_11_, 585.1367).

#### 3.3.3. Rugulosin D (**3**)

Rugulosin D (**3**): Brown amorphous powder; +18.0 (*c* 0.2, MeOH); ^1^H and ^13^C NMR data, Table 2; HRESIMS *m/z* 559.1234 [M + H]^+^ (calcd for C_30_H_23_O_11_, 559.1235).

### 3.4. Antibacterial Activity Assays

The antimicrobial activities were performed based on Antimicrobial Susceptibility Testing Standards outlined by the Clinical and Laboratory Standards Institute document M07-A7 (CLSI) [31] and our previous report [26] by using a penal of pathogens of *C. albicans* ATCC 10231, *S. aureus* ATCC 25923 and *E. coli* ATCC 25922. All the tested compounds were dissolved in dimethyl sulfoxide and diluted in two fold. The minimum inhibitory concentrations (MICs) were determined to be the lowest concentration with no visible bacterial in wells.

## 4. Conclusions

The chemical investigation of a marine-derived fungus *Talaromyces* sp. BTBU20213036 resulted in the isolation of three new compounds (**1**–**3**), and four previously reported metabolites (**4**–**7**). Among them, bacillisporins K and L shared dimeric oligophenaleneone scaffold. Rugulosin D (**4**) is a dimer of the emodin-type anthraquinone. The absolute configurations of isolated compounds were determined by quantum chemical calculations of ECD. Compounds **1**, **2** and **4**−**7** displayed antibacterial activities against *S. aureus* with MIC values of 12.5, 25, 12.5, 6.25, 0.195 and 100 µg/mL, respectively. The difference between **3** and **6** is that the hydroxymethine of C-12′ in **3** was replaced by a sp^2^ quaternary carbon to form an α,β-unsaturated ketene. The α,β-unsaturated ketene moiety enhanced the antibacterial activity of **6** with 64 folds compared to that of **3**. The antibacterial activity of **6** (MIC = 0.195 µg/mL) is much stronger than the positive control of vancomycin (MIC = 1 µg/mL), which indicates it could be considered as a lead compound for further investigations into the mechanism and development of antibacterial agents.

## Figures and Tables

**Table 1:** ^1^H (500 MHz) and ^13^C NMR (125 MHz) data of **1**, **2** and **5** (in DMSO).

Position	1		2		5	
	*δ*_C_	*δ*_H_ (*J* in Hz)	*δ*_C_	*δ*_H_ (*J* in Hz)	*δ*_C_	*δ*_H_ (*J* in Hz)
1	98.8	6.86, s	99.5	6.81, s	165.1	
3	168.3		168.1		164.4	
3a	96.4		96.2		98.4	
3b	130.3		130.2		134.6	
4	162.5		162.4		162.5	
5	118.4	6.96, s	118.8	6.97, s	120.1	7.14, s
6	146.7		146.6		148.1	
6a	118.4		118.4		118.0	
7	139.6		139.8		149.0	
8	134.3		133.8		132.5	
9	152.2		152.4		160.8	
9a	109.0		109.4		100.3	
1′	69.9	5.12, d (12.0) 4.95, d (12.0)	69.7	5.12, d (12.0) 5.00, d (12.0)	69.4	5.18, d (12.5) 5.00, d (12.5)
3′	167.8		167.9		167.6	
3′a	103.7		103.7		104.0	
3′b	147.6		147.8		147.1	
4′	163.1		163.3		163.5	
5′	119.6	6.83, s	119.7	6.83, s	120.1	6.87, s
6′	152.2		152.4		152.5	
6′a	116.6		116.7		116.6	
7′	192.5		192.5		191.2	
8′	64.9	4.83, s	64.6	4.87, d (1.0)	65.5	4.99, s
9′	85.1	4.77, br s	85.6	4.78, d (5.0)	85.2	4.85, s
9′a	49.5		49.7		49.4	
Me-6	24.4	2.98, s	24.6	2.99, s	24.5	3.06, s
Me-6′	23.2	2.48, s	23.2	2.47, s	23.2	2.48, s
1″	78.6	4.12, m	80.2	3.90, m		
2″	68.9	3.72, m	71.3	3.61, m		
3″	15.6	0.75, d (6.5)	18.9	1.14, d (6.5)		
4″	17.2	0.99, d (6.5)	17.9	1.11, d (6.5)		
OH-9′		6.24, d (3.0)		6.31, d (3.0)		

**Table 2:** ^1^H (500 MHz) and ^13^C NMR (125 MHz) NMR data of **3** and **6** (in DMSO).

Position	3		6 [16]	
	*δ*_C_	*δ*_H_ (*J* in Hz)	*δ*_C_	*δ*_H_ (*J* in Hz)
1/1′	178.1/198.8		186.7	
2/2′	55.7/63.4	2.73, d (5.0)/2.90, d (4.5)	59.0	2.77, d (6.0)
3/3′	70.2/69.0	4.27, dd (5.0, 3.0)/4.56, dd (4.5, 4.0)	69.2	4.38, (dd, 6.0, 2.3)
4/4′	48.1/44.0	3.46, brs/3.73, brs	48.4	3.36, brs
5/5′	53.6/63.9		56.3	
6/6′	120.6/120.1	7.46, s/7.41, s	121.2	7.44, d (1.2)
7/7′	148.5/148.6		148.3	
8/8′	124.0/123.8	7.24, s/7.21, s	124.7	7.18, d (1.2)
9/9′	160.9/161.0		160.8	
10/10′	114.3/113.3		114.8	
11/11′	184.8/192.1		181.7	
12/12′	106.8/74.6		106.8	
13/13′	193.0/192.8		194.6	
14/14′	132.3/133.5		132.7	
15/15′	21.6/21.5	2.44, s/2.43, s	22.2	2.41, s
9-OH/	9-OH′	11.71, s/11.04, s		11.4, s

**Table 3:** Antibacterial activity of compounds **1**–**7** (MIC, µg/mL).

Number	1	2	3	4	5	6	7	Control
*C. albicans*	>100	>100	>100	>100	>100	>100	>100	1 ^a^
*S. aureus*	12.5	25	>100	12.5	6.25	0.195	100	1 ^b^
*E. coli*	>100	>100	>100	>100	>100	>100	>100	1 ^c^

^a^ Rapamycin, ^b^ Vancomycin, ^c^ Ciprofloxacin.

## Data Availability

Data are contained within the text.

## Supplementary Materials

The following are available online at https://www.mdpi.com/article/10.3390/antibiotics11020222/s1, Figures S1–S24: HRESIMS, HPLC profiles, 1D and 2D NMR spectra for compounds **1**–**3**, Figures S25 and S26: Colony morphology and Neighbor-joining phylogenetic tree of strain BTBU20213036, Figure S27: Flow chart of the fermentation, extraction and isolation, Tables S1–S3: 1D and 2D NMR data for compounds **1**–**3**.
