# Supplementary material for: New Antibacterial Secondary Metabolites from a Marine-Derived Talaromyces sp. Strain BTBU20213036
Source: Antibiotics (Basel). 2022 Feb 10;11(2):222. doi: 10.3390/antibiotics11020222 (PMC8868179; doi:10.3390/antibiotics11020222)
Supplement: Supplementary file 1 [file antibiotics-11-00222-s001.zip › antibiotics-1576341-supplementary.pdf]

SUPPLEMENTARY MATERIAL

New antibacterial secondary metabolites from a marine-derived *Talaromyces* sp. strain BTBU20213036

Fuhang Song [1], Yifei Dong [1], Shangzhu Wei [2], Xinwan Zhang [2], Kai Zhang [1] and Xiuli Xu [2,*]

Table of Contents

Figure S1. HRESIMS spectrum for **1** 3
Figure S2. HPLC profile and UV spectrum for **1** 3
Figure S3. ^{1}H NMR spectrum (500 MHz, DMSO-d_6) of **1** 4
Figure S4. ^{13}C NMR spectrum (125 MHz, DMSO-d_6) of **1** 4
Figure S5. HSQC spectrum (500 MHz, DMSO-d_6) of **1** 5
Figure S6. ^{1}H -^{1}H COSY spectrum (500 MHz, DMSO-d_6) of **1** 5
Figure S7. HMBC spectrum (500 MHz, DMSO-d_6) of **1** 6
Figure S8. ROESY spectrum (500 MHz, DMSO-d_6) of **1** 6
Figure S9. HRESIMS spectrum for **2** 7
Figure S10. HPLC profile and UV spectrum for **2** 7
Figure S11. ^{1}H NMR spectrum (500 MHz, DMSO-d_6) of **2** 8
Figure S12. ^{13}C NMR spectrum (125 MHz, DMSO-d_6) of **2** 8
Figure S13. HSQC spectrum (500 MHz, DMSO-d_6) of **2** 9
Figure S14. ^{1}H -^{1}H COSY spectrum (500MHz, DMSO-d_6) of **2** 9
Figure S15. HMBC spectrum (500MHz, DMSO) of **2** 10
Figure S16. ROESY spectrum (500 MHz, DMSO-d_6) of **2** 10
Figure S17. HRESIMS spectrum for **3** 11
Figure S18. HPLC profile and UV spectrum for **3** 11
Figure S19. ^{1}H NMR spectrum (500 MHz, DMSO-d_6) of **3** 12
Figure S20. ^{13}C NMR spectrum (125 MHz, DMSO-d_6) of **3** 12
Figure S21. HSQC spectrum (500 MHz, DMSO-d_6) of **3** 13
Figure S22. ^{1}H -^{1}H COSY spectrum (500 MHz, DMSO-d_6) of **3** 13
Figure S23. HMBC spectrum (500 MHz, DMSO-d_6) of **3** 14
Figure S24. ROESY spectrum (500 MHz, DMSO-d_6) of **3** 14
Figure S25. Colony Morphology of strain BTBU20213036 15
Figure S26. Neighbor-joining phylogenetic tree of strain BTBU20213036 16
Figure S27. Flow chart of the fermentation, extraction and isolation 17
Table S1. 1D and 2D NMR data for **1** 18
Table S2. 1D and 2D NMR data for **2** 19
Table S3. 1D and 2D NMR data for **3** 20

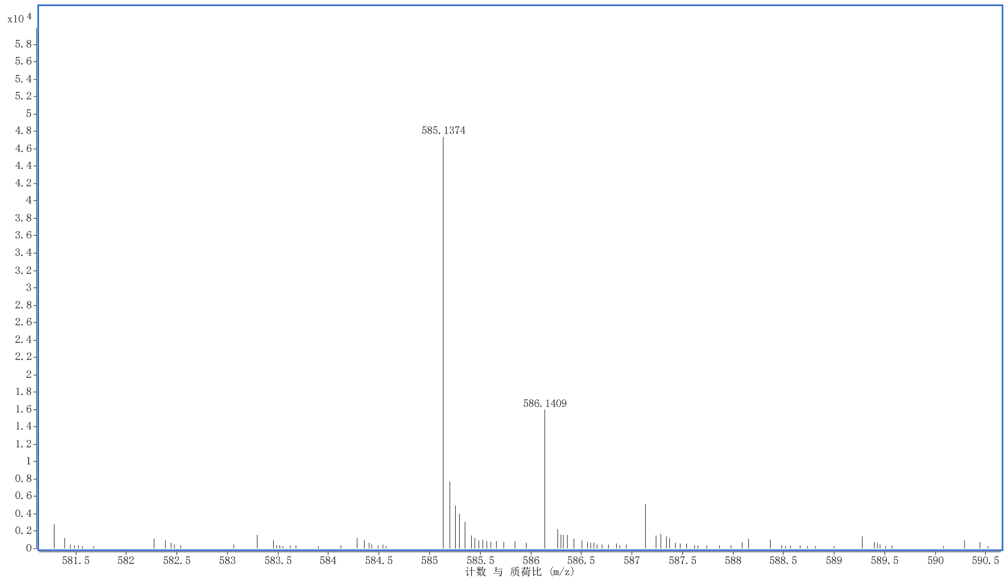

Figure S1. HRESIMS spectrum for **1**

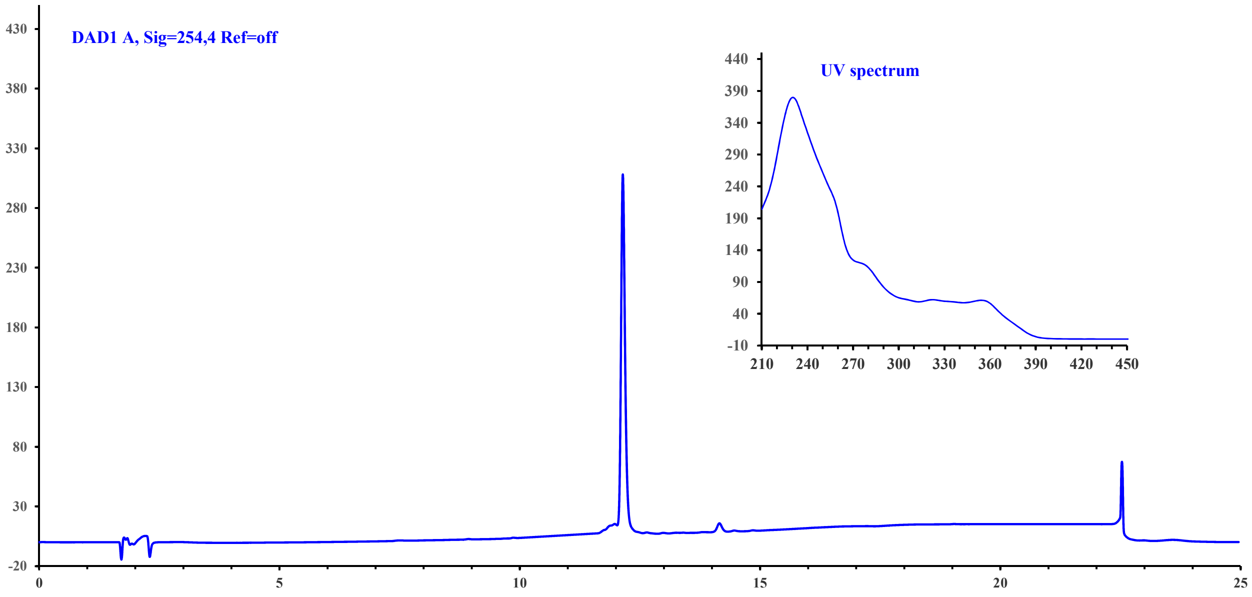

Figure S2. HPLC profile and UV spectrum for **1**

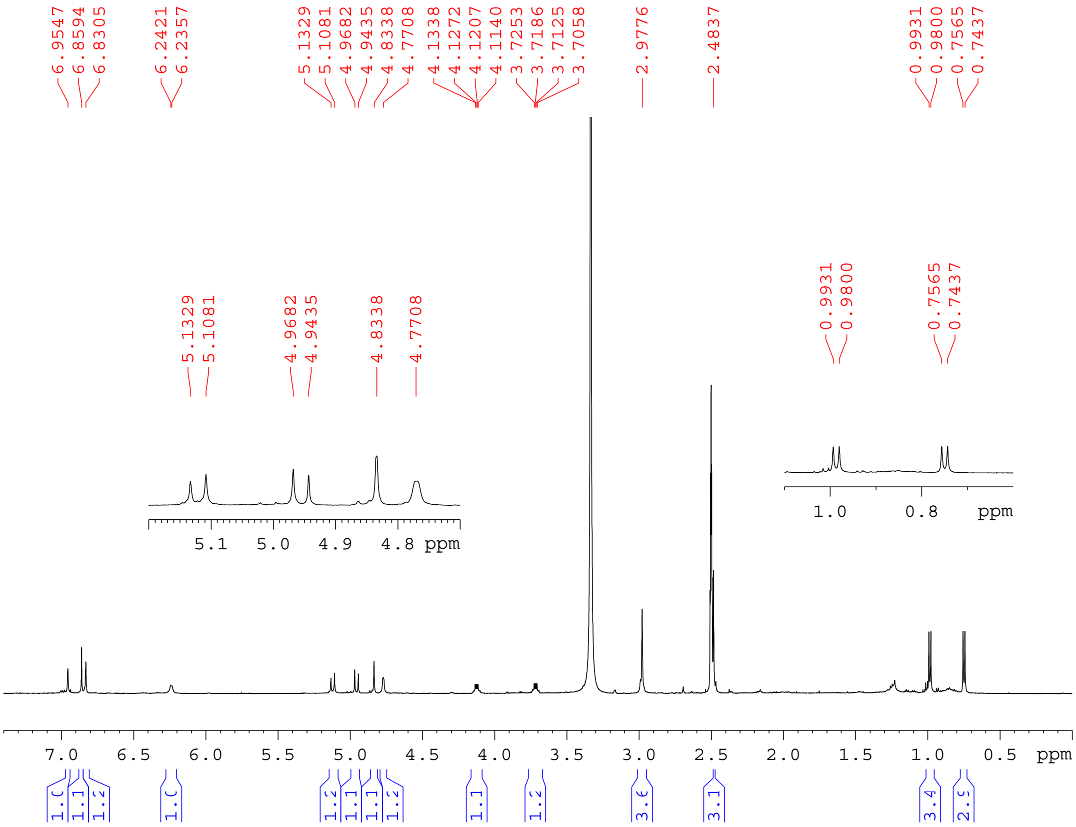

Figure S3. ^{1}H NMR spectrum (500 MHz, DMSO-d_6) of **1**

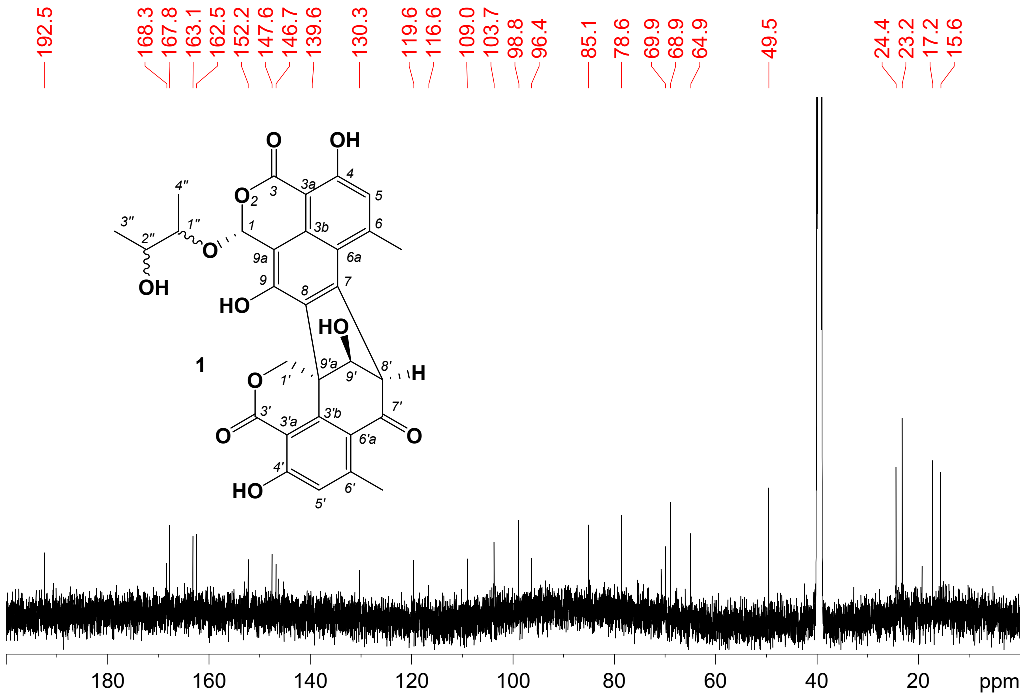

Figure S4. ^{13}C NMR spectrum (125 MHz, DMSO-d_6) of **1**

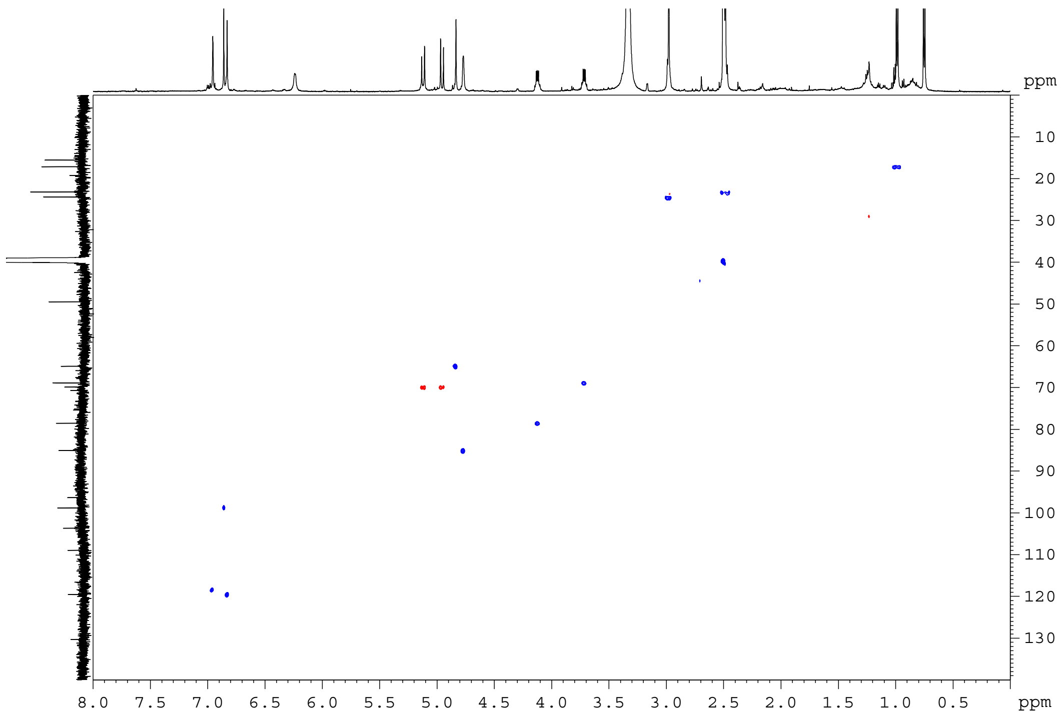

Figure S5. HSQC spectrum (500 MHz, DMSO-d_6) of **1**

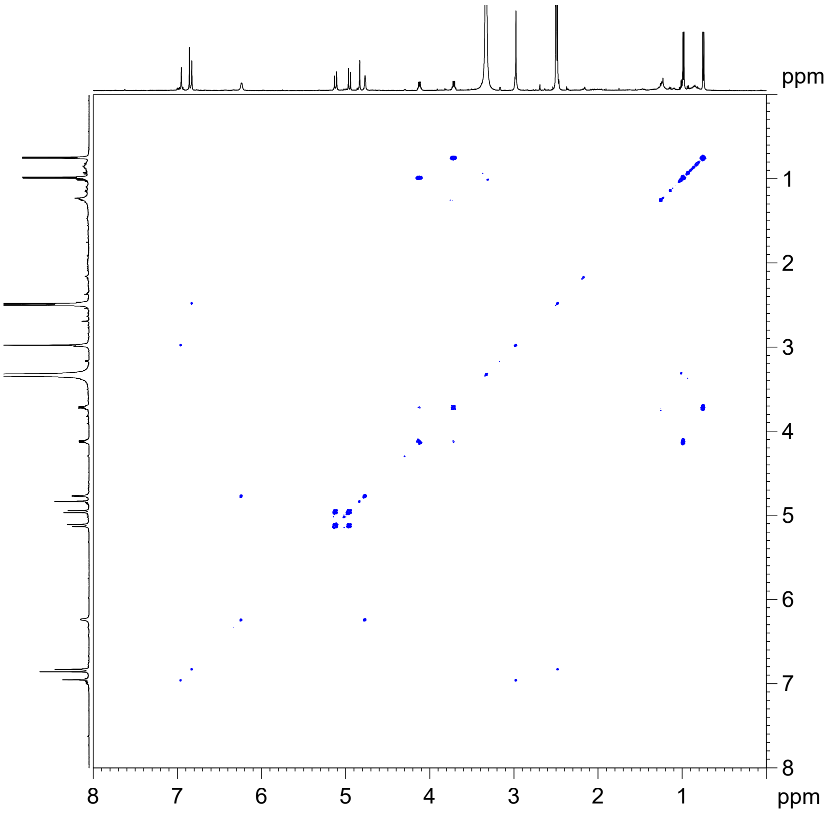

Figure S6. ^{1}H -^{1}H COSY spectrum (500MHz, DMSO-d_6) of **1**

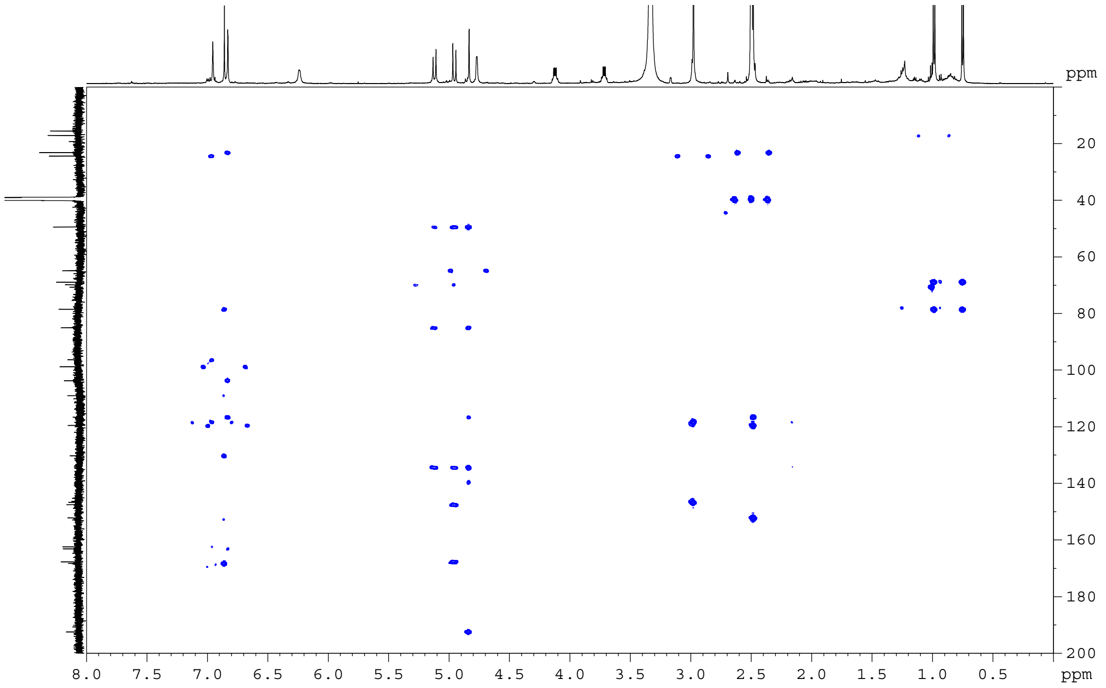

Figure S7. HMBC spectrum (500 MHz, DMSO-d_6) of **1**

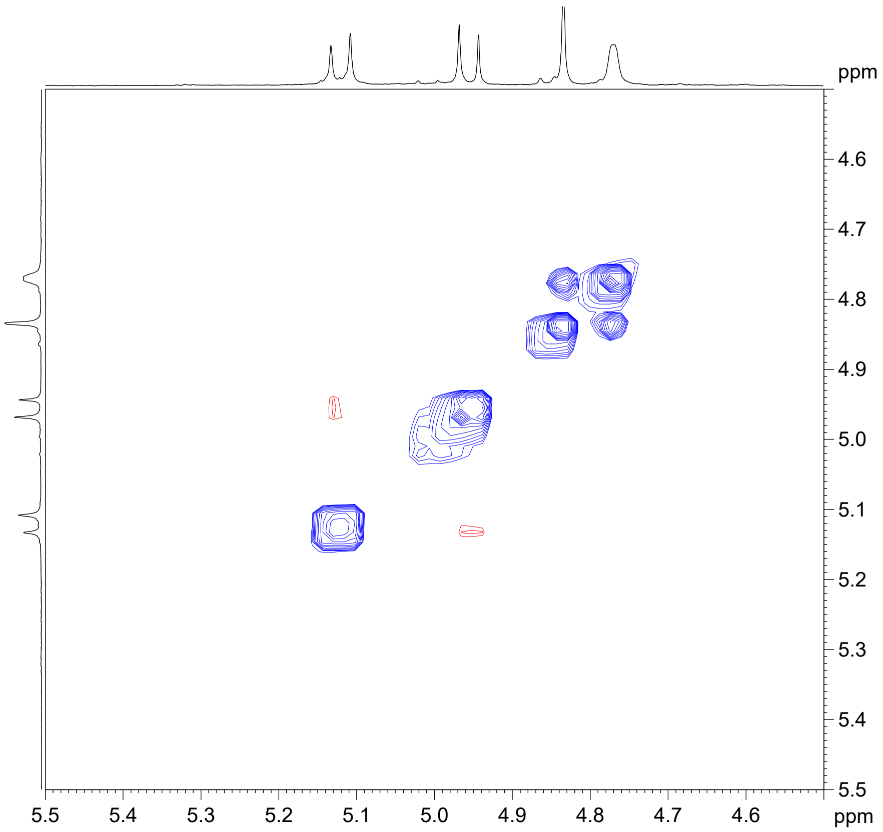

Figure S8. ROESY spectrum (500 MHz, DMSO-d_6) of **1**

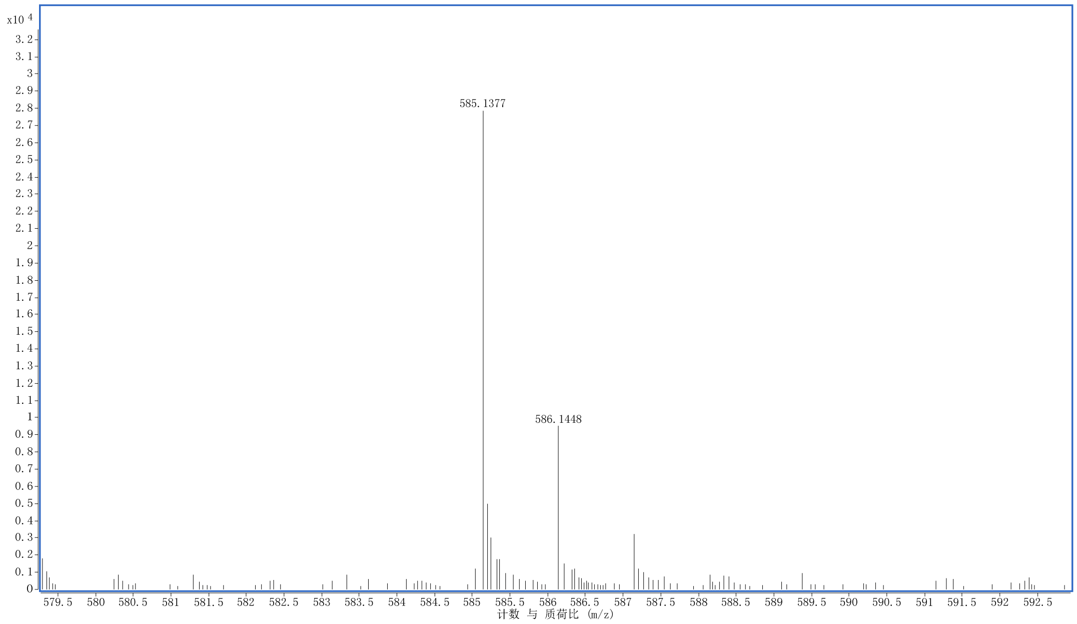

Figure S9. HRESIMS spectrum for **2**

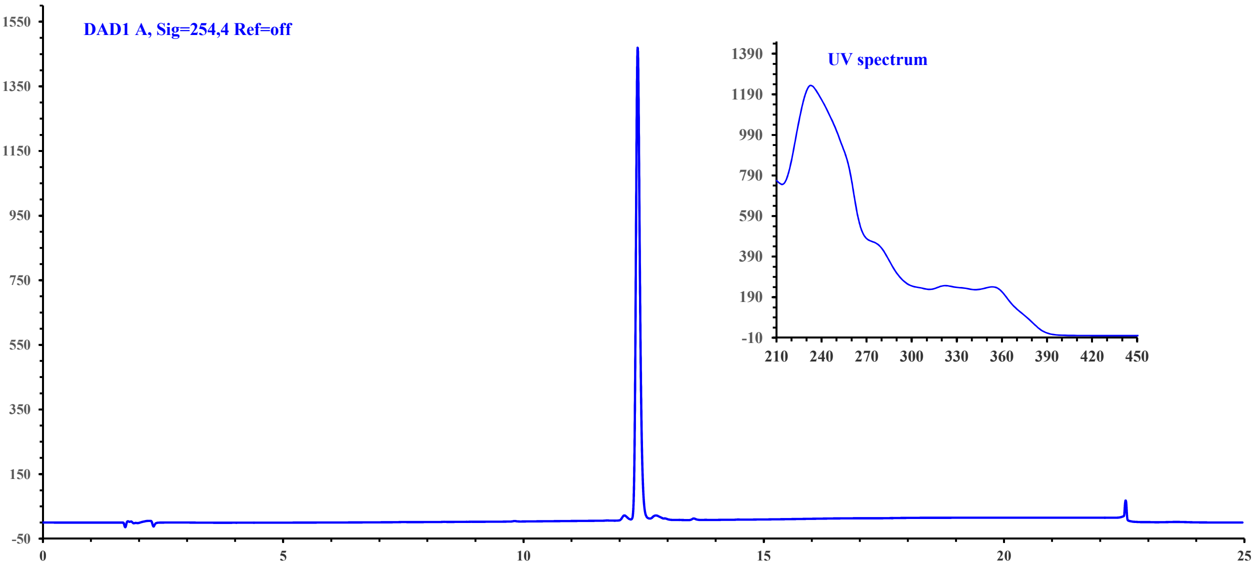

Figure S10. HPLC profile and UV spectrum for **2**

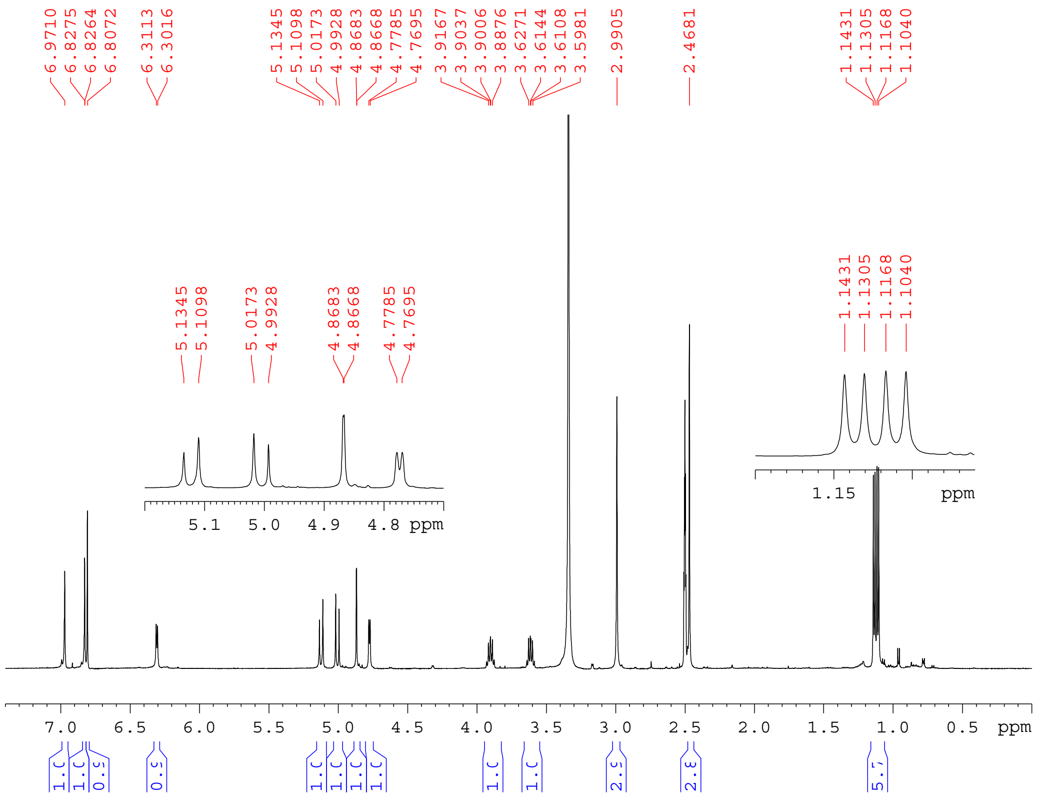

Figure S11. ^{1}H NMR spectrum (500 MHz, DMSO-d_6) of **2**

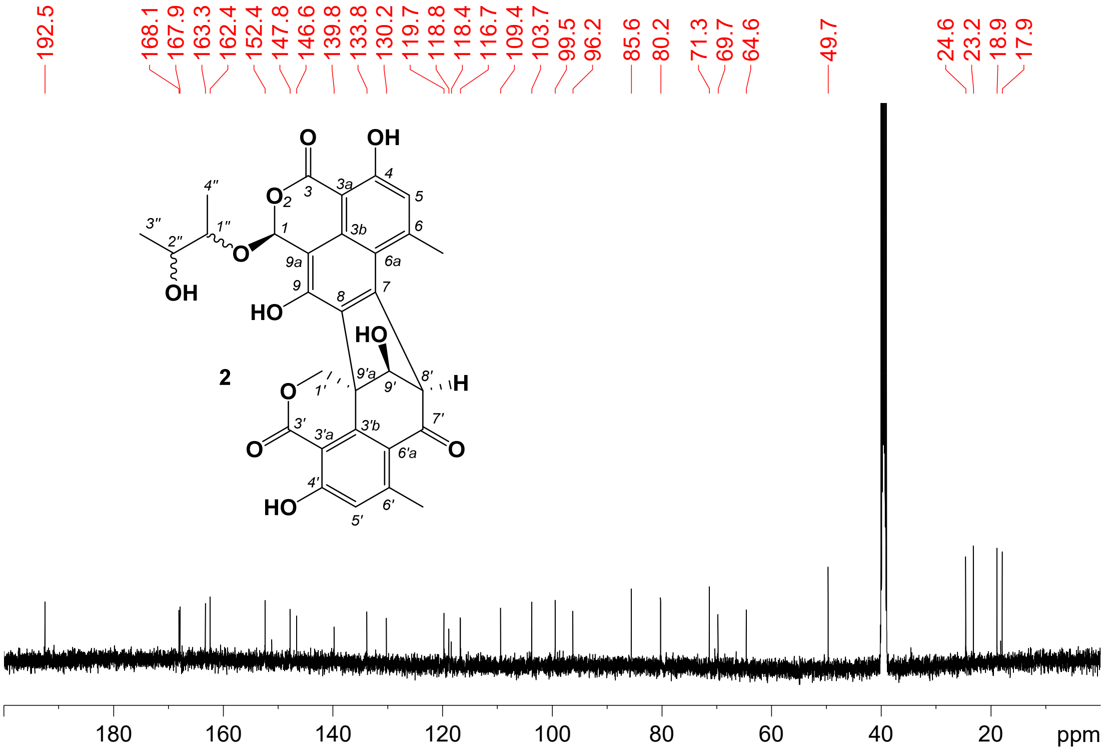

Figure S12. ^{13}C NMR spectrum (125 MHz, DMSO-d_6) of **2**

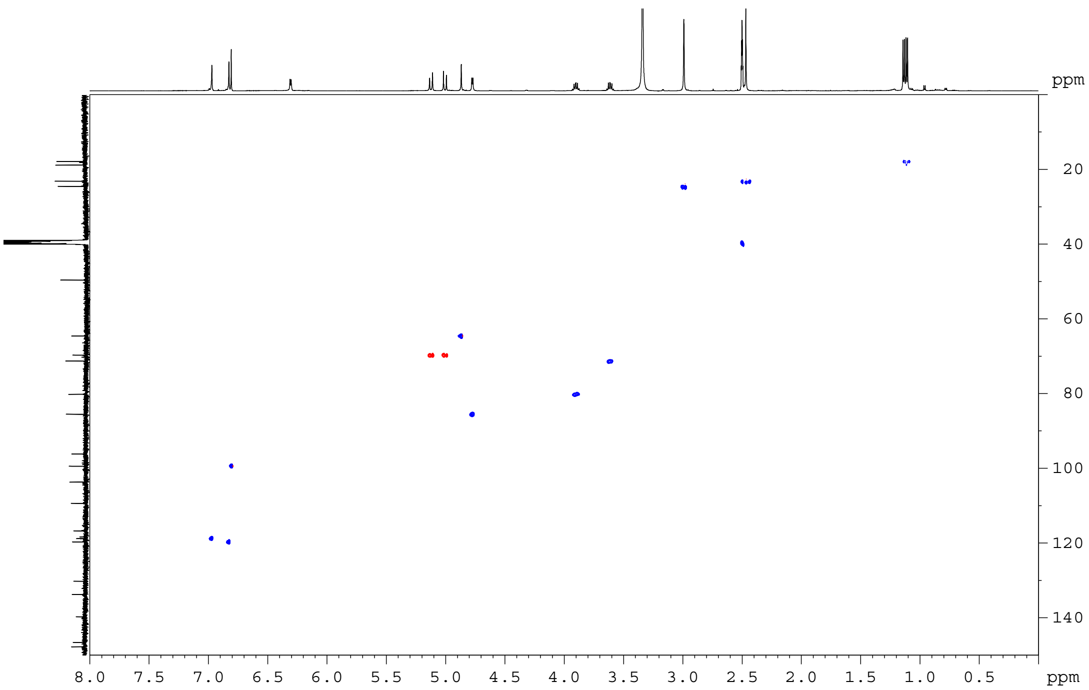

Figure S13. HSQC spectrum (500 MHz, DMSO-d_6) of **2**

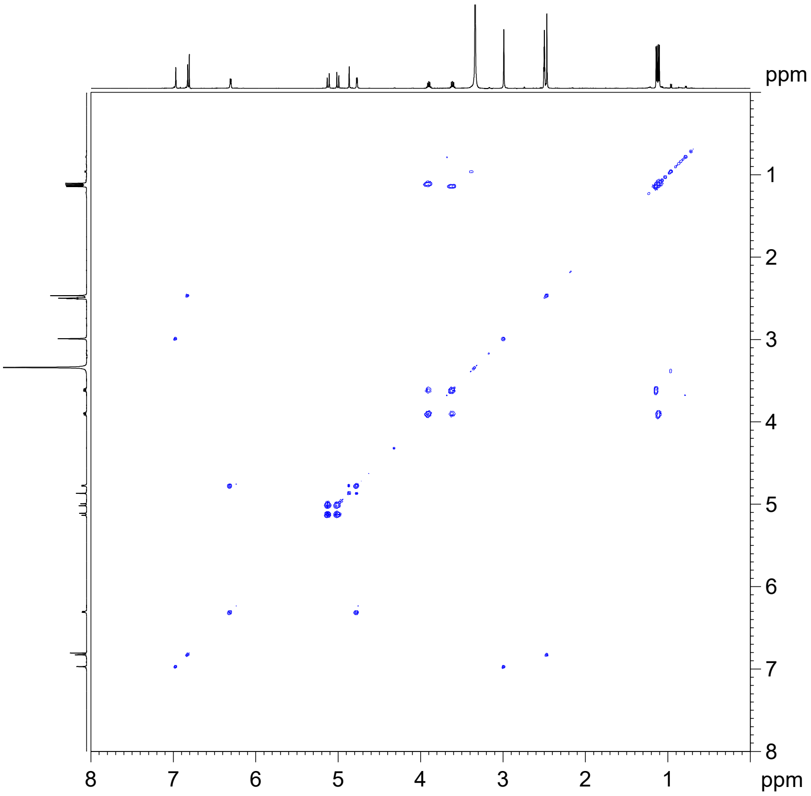

Figure S14. ^{1}H -^{1}H COSY spectrum (500 MHz, DMSO-d_6) of **2**

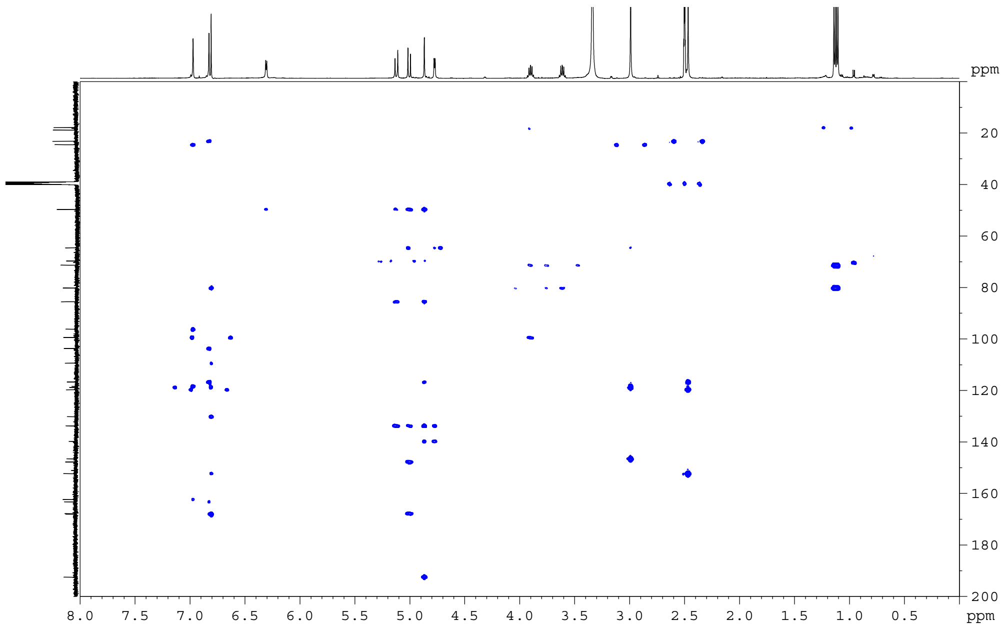

Figure S15. HMBC spectrum (500 MHz, DMSO-d_6) of **2**

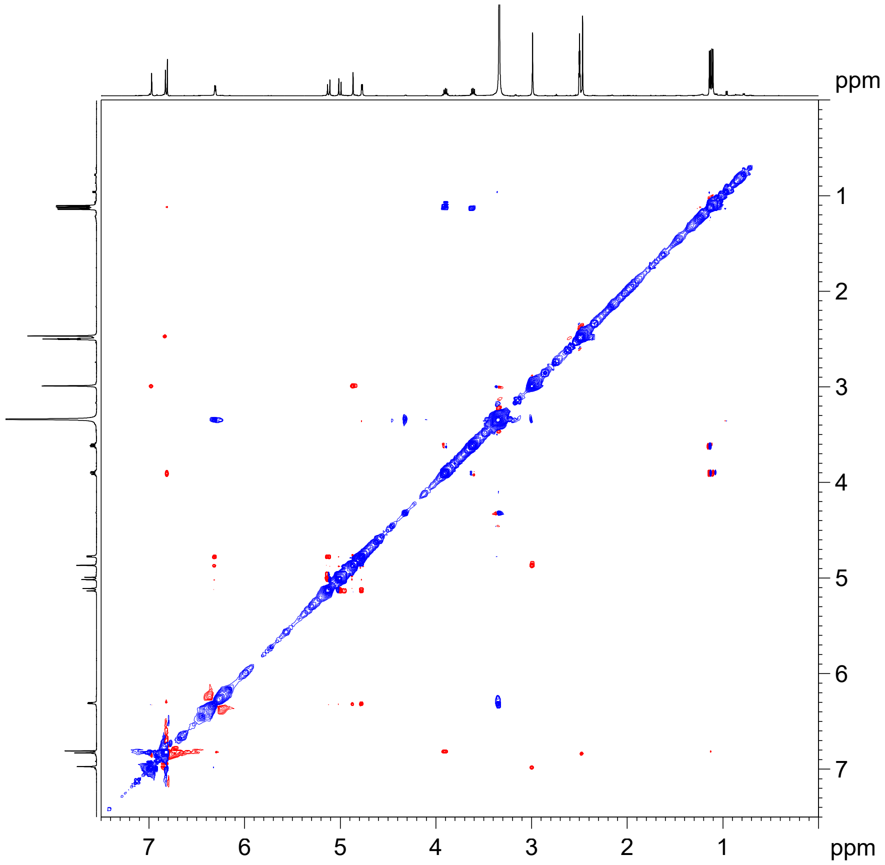

Figure S16. ROESY spectrum (500 MHz, DMSO-d_6) of **2**

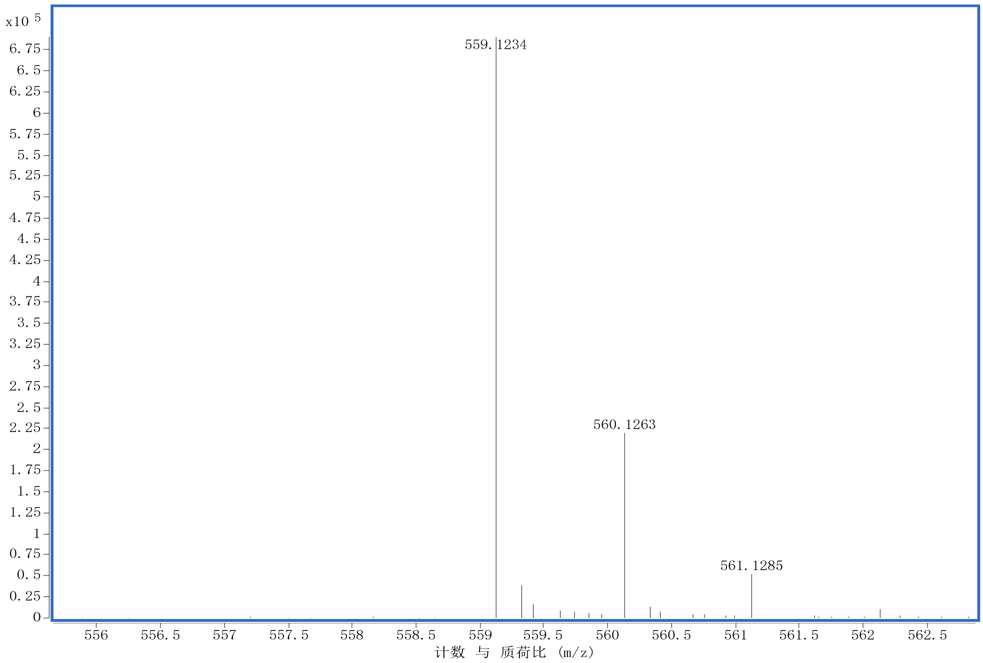

Figure S17. HRESIMS spectrum for **3**

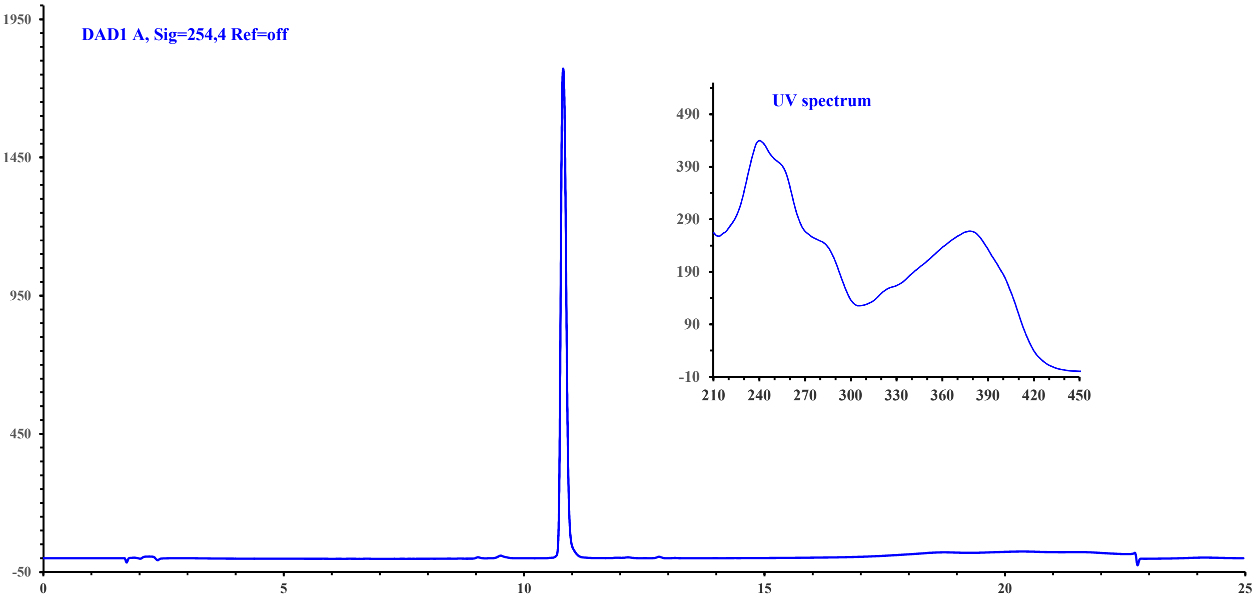

Figure S18. HPLC profile and UV spectrum for **3**

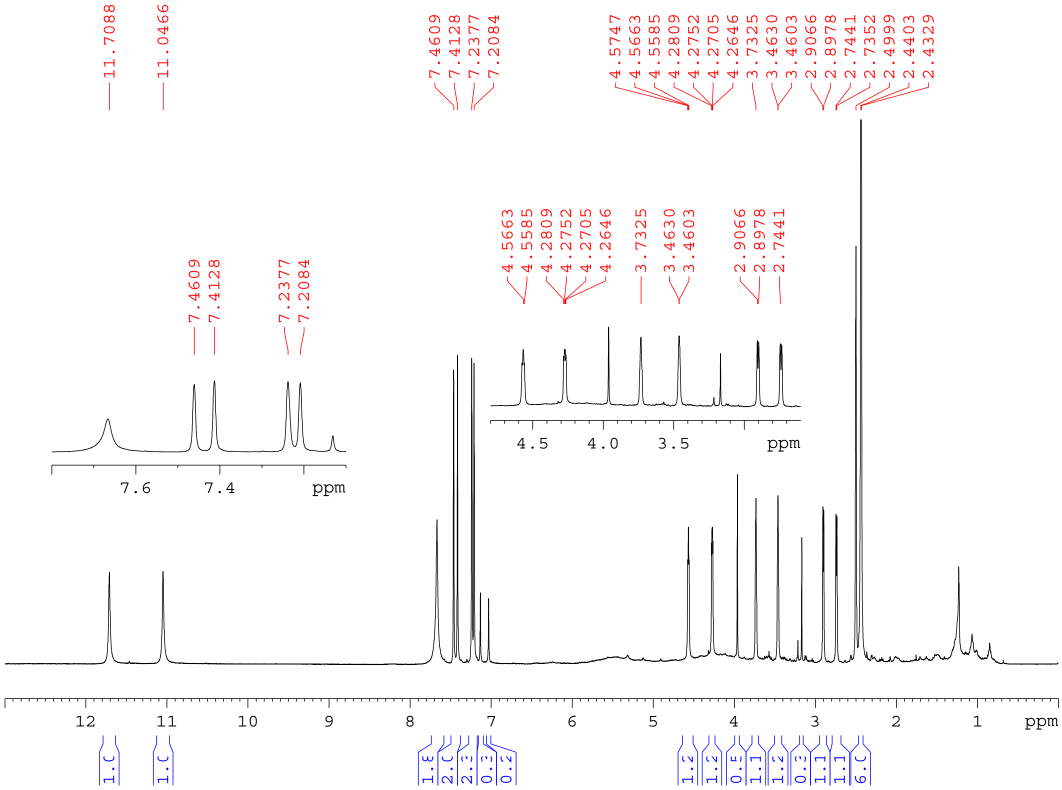

Figure S19. ^{1}H NMR spectrum (500 MHz, DMSO-d_6) of **3**

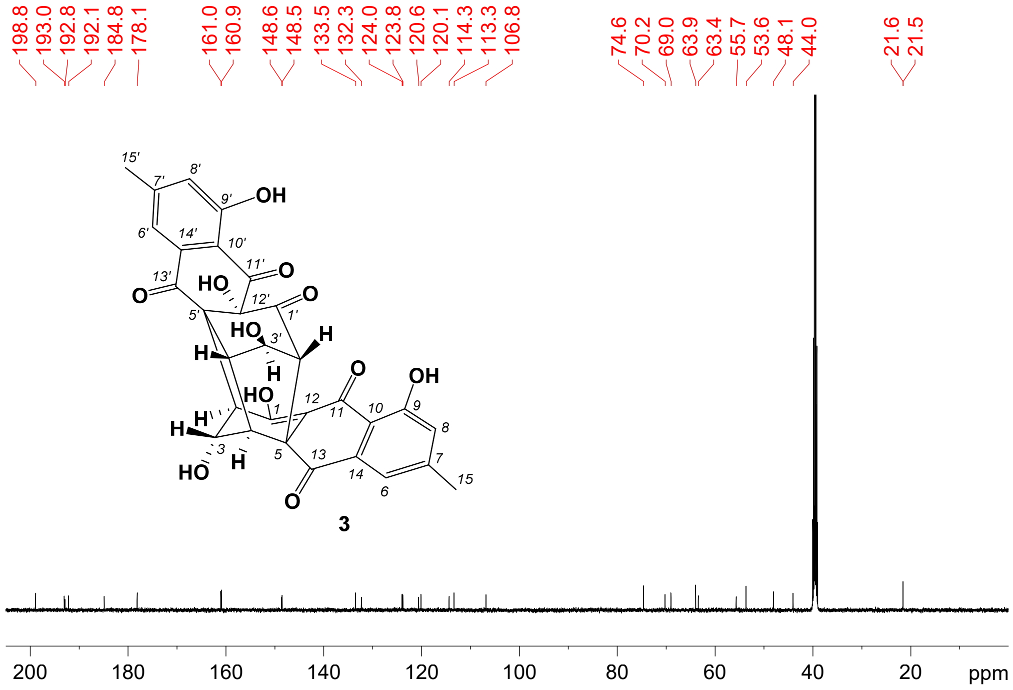

Figure S20 ^{13}C NMR spectrum (125 MHz, DMSO-d_6) of **3**

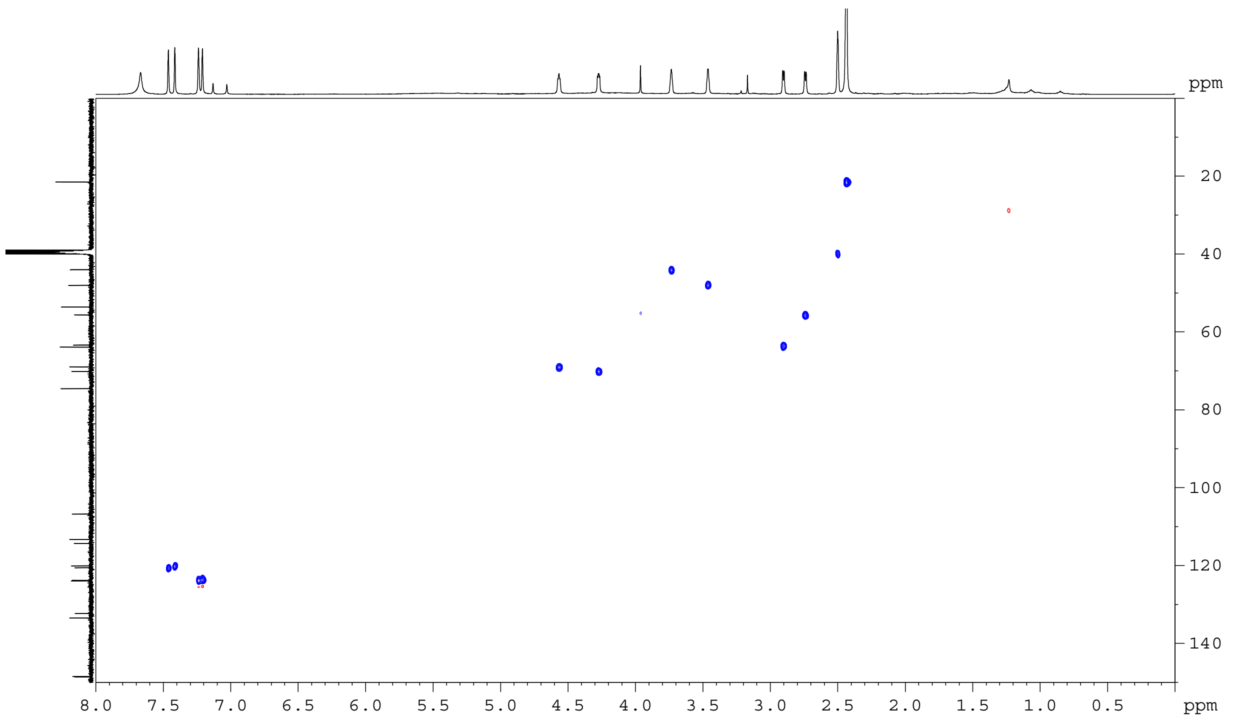

Figure S21. HSQC spectrum (500 MHz, DMSO-d_6) of **3**

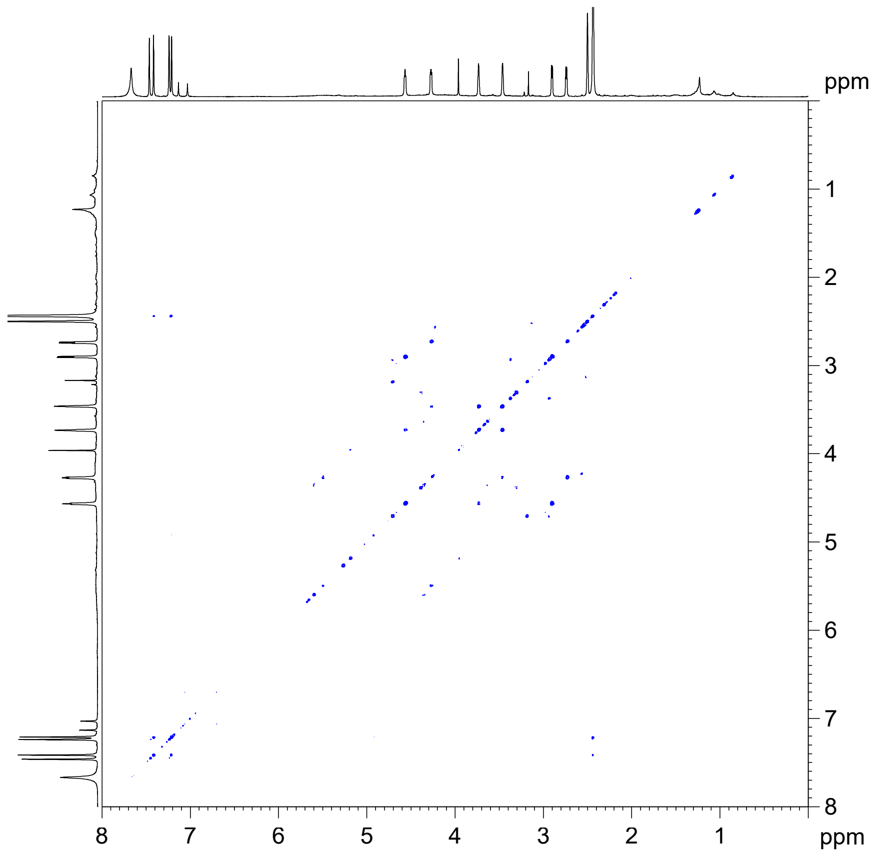

Figure S22. ^{1}H -^{1}H COSY spectrum (500 MHz, DMSO-d_6) of **3**

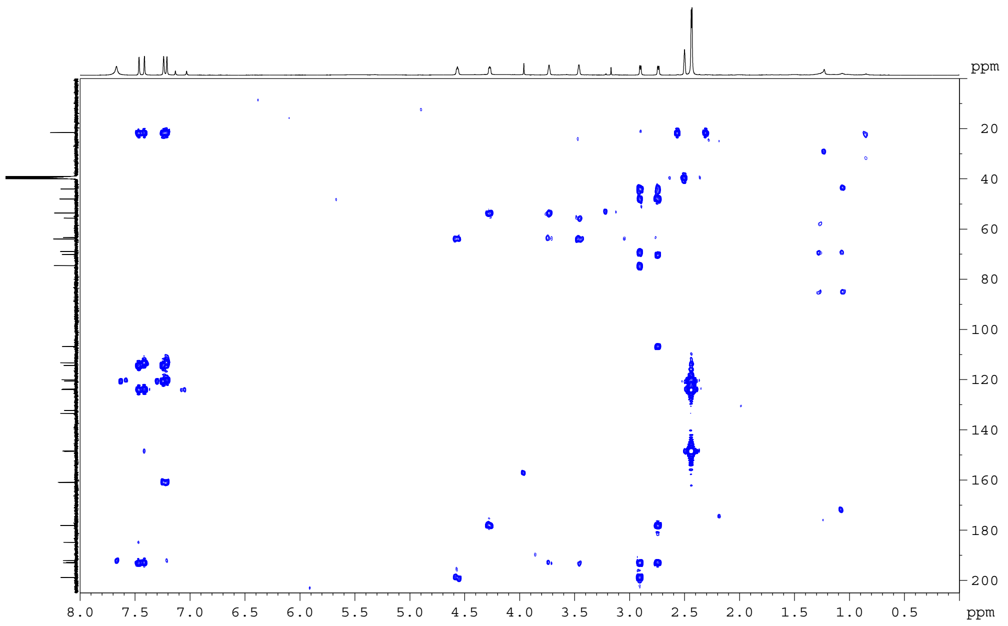

Figure S23. HMBC spectrum (500 MHz, DMSO-6) of **3**

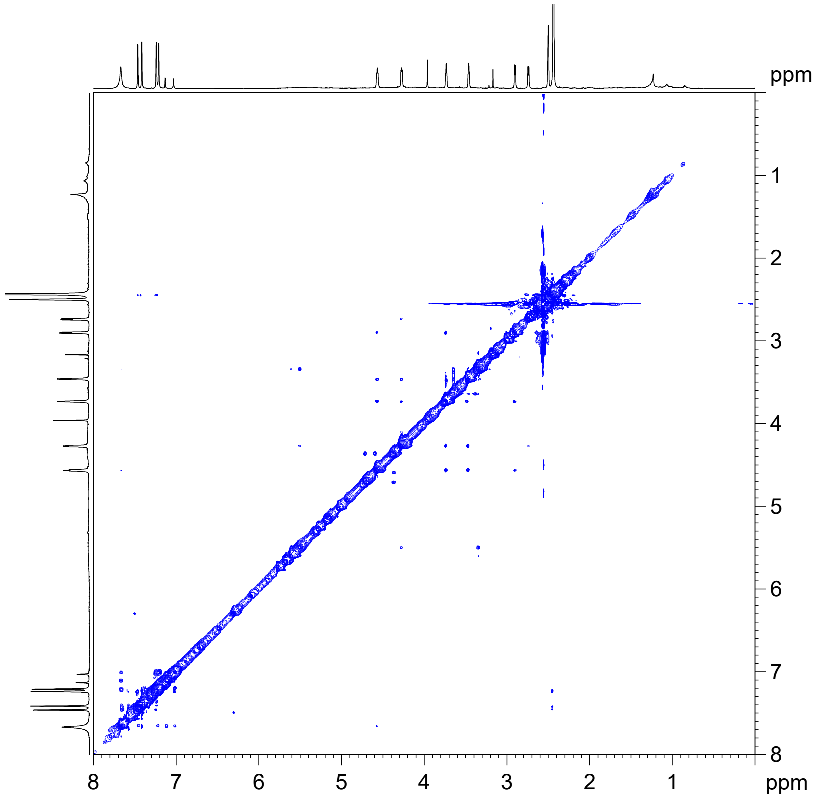

Figure S24. ROESY spectrum (500 MHz, DMSO-6) of **3**

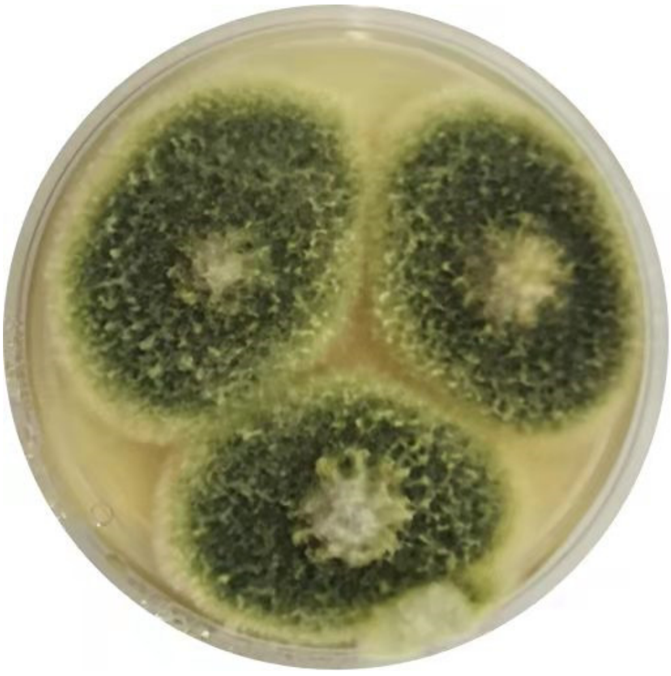

Figure S25. Colony Morphology of strain BTBU20213036

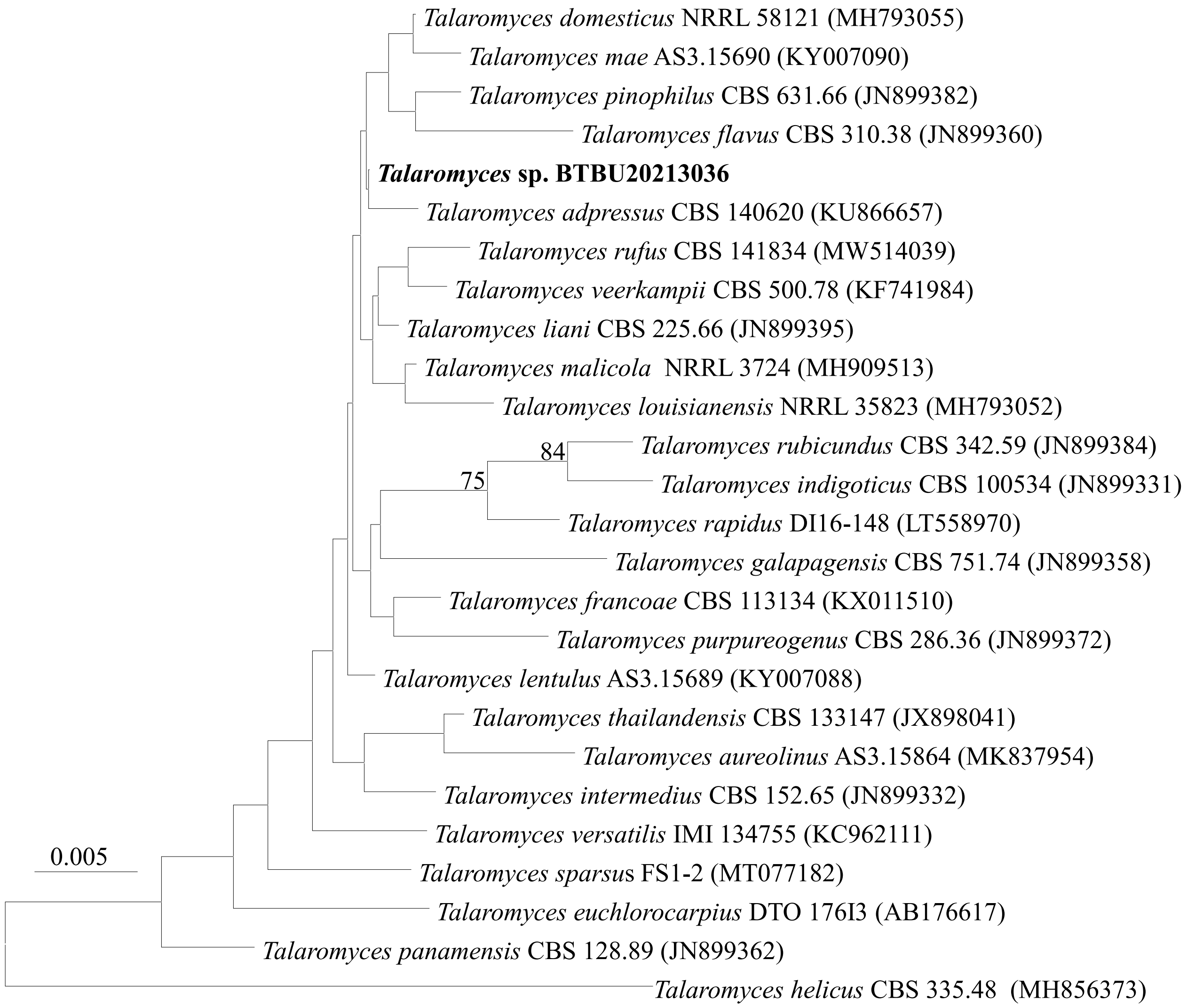

Figure S26. The Neighbor-joining phylogram inferred from the ITS sequences. Percentages over 70% derived from 1000 replicates are indicated at the nodes, the strain in this study is indicated in boldface. — Bar = 0.005 substitutions per nucleotide position.

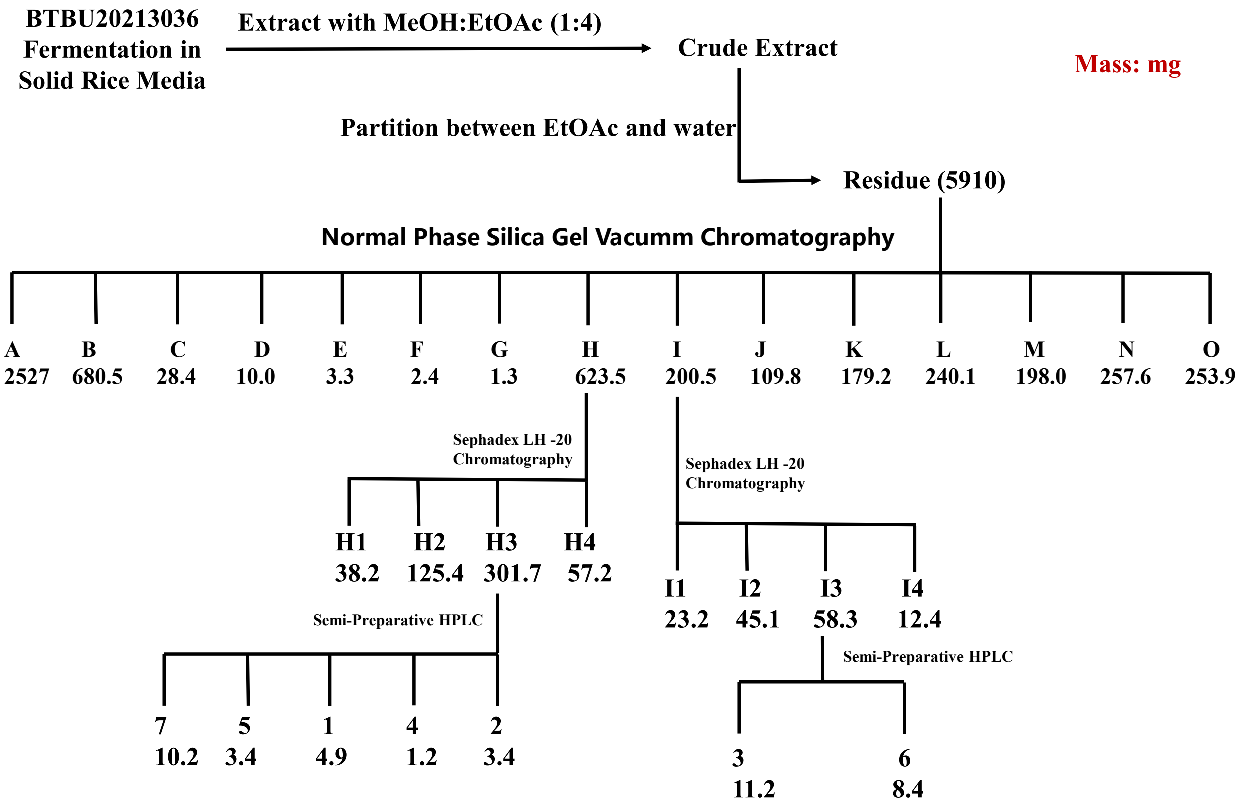

Figure S27. Flow chart of the fermentation, extraction and isolation

Table S1. ^{1}H (500 MHz), ^{13}C NMR (125 MHz), HMBC and ROESY correlations of **1** (in DMSO).

Position	δC	δH (*J* in Hz)	HMBC (H→C)	ROESY
1	98.8	6.86, s	3, 3b, 9, 9a, 1''	
3	168.3			
3a	96.4			
3b	130.3			
4	162.5			
5	118.4	6.96, s	3a, 4, 5, Me-6	
6	146.7			
6a	118.4			
7	139.6			
8	134.3			
9	152.2			
9a	109.0			
1'	69.9	5.12, d (12.0)<br>4.95, d (12.0)	8, 9', 9'a<br>8, 3', 3'b, 9'a	
3'	167.8			
3'a	103.7			
3'b	147.6			
4'	163.1			
5'	119.6	6.83, s	3'a, 4', 6'a, Me-6'	
6'	152.2			
6'a	116.6			
7'	192.5			
8'	64.9	4.83, s	7, 8, 6'a, 7', 9', 9'a	9'
9'	85.1	4.77, br s		8'
9'a	49.5			
Me-6	24.4	2.98, s	5, 6, 6a	
Me-6'	23.2	2.48, s	5', 6', 6'a	
1''	78.6	4.12, m	1	
2''	68.9	3.72, m		
3''	15.6	0.75, d (6.5)	1'', 2''	
4''	17.2	0.99, d (6.5)	1'', 2''	
OH-9'		6.24, d (3.0)		

Table S2. ^{1}H (500 MHz), ^{13}C NMR (125 MHz), HMBC and ROESY correlations of **2** (in DMSO).

Position	δ_C	δ_H (J in Hz)	HMBC (H→C)	ROESY
1	99.5	6.81, s	3, 3b, 9, 9a, 1''	
3	168.1			
3a	96.2			
3b	130.2			
4	162.4			
5	118.8	6.97, s	3a, 4, 6a, Me-6	
6	146.6			
6a	118.4			
7	139.8			
8	133.8			
9	152.4			
9a	109.4			
1'	69.7	5.12, d (12.0)<br>5.00, d (12.0)	8, 9', 9'a<br>8, 3', 3'b, 9'a	9'
3'	167.9			
3'a	103.7			
3'b	147.8			
4'	163.3			
5'	119.7	6.83, s	3'a, 4', 6', Me-6'	
6'	152.4			
6'a	116.7			
7'	192.5			
8'	64.6	4.87, d (1.0)	7, 8, 6'a, 7', 9', 9'a	OH-9'
9'	85.6	4.78, d (5.0)	7, 8	1'a
9'a	49.7			
Me-6	24.6	2.99, s	5, 6, 6a	
Me-6'	23.2	2.47, s	5', 6', 6'a	
1''	80.2	3.90, m		
2''	71.3	3.61, m		
3''	18.9	1.14, d (6.5)	1'', 2''	
4''	17.9	1.11, d (6.5)	1'', 2''	
OH-9'		6.31, d (3.0)	9'a	8'

Table S3. ^{1}H (500 MHz), ^{13}C NMR (125 MHz), HMBC and ROESY correlations of **3** (in DMSO).

Position	δ_C	δ_H (J in Hz)	HMBC (H→C)	ROESY
1	178.1			
2	55.7	2.73, d (5.0)	1, 3, 4, 4', 13'	3
3	70.2	4.27, dd (5.0, 3.0)	1, 5	2, 4'
4	48.1	3.46, brs	5, 2'	3'
5	53.6			
6	120.6	7.46, s	8, 10, 13, 15	
7	148.5			
8	124.0	7.24, s	6, 9, 10, 15	
9	160.9			
10	114.3			
11	184.8			
12	106.8			
13	193.0			
14	132.3			
15	21.6	2.44, s	6, 7, 8	
9-OH		11.71, s		
1'	198.8			
2'	63.4	2.90, d (4.5)	4, 13, 1', 3', 4', 12'	3'
3'	69.0	4.56, dd (4.5, 4.0)	1', 5'	4, 2', 4'
4'	44.0	3.73, brs	5	3, 3'
5'	63.9			
6'	120.1	7.41, s	8', 10', 13', 15'	
7'	148.6			
8'	123.8	7.21, s	6', 9', 10', 15'	
9'	161.0			
10'	113.3			
11'	192.1			
12'	74.6			
13'	192.8			
14'	133.5			
15'	21.5	2.43, s	6', 7', 8'	
9-OH'		11.04, s		
